# Iron-Deficiency Anemia in Chronic Kidney Disease: A Literature Review of Its Pathophysiology, Diagnosis, and Management

**DOI:** 10.7759/cureus.77598

**Published:** 2025-01-17

**Authors:** Saba Kazmi, Viktoriia Zarovniaeva, Kimberly Cortez Perez, Sehej Sandhu, Summayya Anwar, Lubna Mohammed

**Affiliations:** 1 Internal Medicine, California Institute of Behavioral Neurosciences & Psychology, Fairfield, USA; 2 Radiology, California Institute of Behavioral Neurosciences & Psychology, Fairfield, USA; 3 Biosciences, COMSATS (Commission on Science and Technology for Sustainable Development in the South) University Islamabad, Islamabad, PAK

**Keywords:** chronic kidney disease (ckd), erythropoietin (epo), hemoglobin: hb, iron-deficiency, iron deficiency anemia (ida)

## Abstract

Anemia is a prevalent complication of chronic kidney disease (CKD) and is associated with increased mortality and reduced health-related quality of life. The iron-rich protein hemoglobin, which the body utilizes to carry oxygen, deteriorates in anemia. Because hemoglobin is a protein that contains iron, disturbances in iron homeostasis can lead to iron-deficiency anemia (IDA). CKD frequently results in anemia, which is linked to a poor prognosis. Nevertheless, whether anemia per se or other comorbidities are the reason for this connection remains unclear. Uncertainty surrounds whether distinct forms of IDA can forecast CKD outcomes. Physicians, nurse practitioners, physician assistants, and registered nurses work as a team to manage anemia in patients with CKD. Individuals with CKD can benefit from the involvement of different specialties along the treatment continuum, and dietitians/nutritionists can play a significant role in enhancing management through multidisciplinary care. This literature review aims to deliver a comprehensive overview of IDA in CKD, concentrating on its diagnosis. It addresses the pathophysiology, complications associated with iron deficiency, and the available diagnostic tests for IDA in CKD. Furthermore, we analyze the literature that has contributed to developing current practice guidelines for treating IDA in CKD and encouraging multidisciplinary team collaboration in managing IDA in CKD.

## Introduction and background

Anemia is a medical condition marked by low hemoglobin (Hgb) levels, which are crucial for the transportation of oxygen in red blood cells (RBCs) and are an iron-rich protein [1-3]. Anemia is defined by the World Health Organization (WHO) as having a Hgb concentration of less than 12 g/dl in non-pregnant women and less than 13 g/dl in adult males. The incidence of anemia rises as chronic kidney disease (CKD) progresses, with estimates ranging from 7% to over 50% in the later stages. Various factors contribute to anemia in CKD, with the most significant being a relative shortage of erythropoietin (EPO). Consequently, erythropoiesis-stimulating agents (ESAs) have become a key part of managing anemia in CKD patients. However, several well-designed studies have shown that using ESAs to achieve normal Hgb levels in CKD patients may lead to worse cardiovascular outcomes [3]. As a result, current guidelines recommend aiming for a Hgb target that is below the normal range for individuals with CKD.

A CKD diagnosis requires two criteria: a glomerular filtration rate (GFR) of less than 60 ml/min/1.73 m^2^ or evidence of kidney damage, such as albuminuria. CKD is defined by abnormalities in kidney structure or function that last more than three months and can have severe health consequences. Given that comorbid conditions in CKD patients are likely to increase mortality rates, CKD presents a significant public health issue. Anemia is a common complication of CKD, impacting both patients and healthcare systems financially. In the later stages of CKD, particularly in stage G4 (with an estimated GFR of 15 to 30 ml/min), anemia becomes increasingly prevalent, affecting most patients. Iron deficiency in CKD patients has a variety of causes. As kidney function declines, the prevalence of anemia rises. Diabetes mellitus patients have worse anemia for any given eGFR severity [3]. In CKD, anemia has a complex etiology [4], with relative EPO insufficiency being widespread. Additionally, several other factors reduce the marrow's sensitivity to EPO, including inflammation and iron deficiency, which are the two most prevalent causes. Many patients with anemia and CKD develop iron-deficiency anemia (IDA), which can result from either a relative or functional iron deficiency or from a true absence of iron reserves (absolute IDA). The latter is typically caused by underlying inflammation, which impairs the body's ability to utilize stored iron [5].

This literature review aims to deliver a comprehensive overview of IDA in CKD, concentrating on its diagnosis. It addresses the mechanism of IDA, complications associated with iron deficiency, and the available diagnostic tests for IDA in CKD. Furthermore, we analyze the literature that has contributed to developing current practice guidelines for treating IDA in CKD.

## Review

Search strategy and databases

In our search data on the prevalence of IDA in individuals with CKD, we accessed multiple databases, including PubMed Central, Google Scholar, and PubMed. We used specific keywords such as chronic kidney disease (CKD), iron-deficiency anemia (IDA), hemoglobin, erythropoietin (EPO), and iron. To enhance efficiency, we utilized the EndNote reference manager to organize the references and remove duplicates. We then screened the studies for the last 15 years (2009-2024) by reviewing their titles and abstracts to eliminate irrelevant ones. Finally, we conducted a detailed review of the full-text papers to assess their quality. Every co-author in the literature review contributed at a different level, so some helped with data selection, grammar corrections, additions or deletions, and oversight of each review phase. To talk about the literature review's progress and potential improvements, we had weekly Zoom meetings. A final draft of the review was created through eight reunions, during which all participants provided their final comments on the article's substance and evaluation of the findings before submission. This study's primary outcome focused on the prevalence of IDA in individuals with CKD, concentrating on its diagnosis, current practice guidelines for treating IDA in CKD, and encouraging multidisciplinary team collaboration in managing IDA in CKD.

Discussion

Mechanism of IDA

The life cycle of RBCs involves tightly regulated iron metabolism at several stages. The differentiation of erythroblasts into reticulocytes relies on iron, while the development of erythroid lineage from multipotent myeloid stem cells is controlled by EPO. Consequently, insufficient iron can impair the body's response to EPO [6,7]. During iron absorption in the intestines, iron binds to serum transferrin, after which it is directed either to the bone marrow for RBC production or to the liver and spleen for storage, where it is associated with ferritin [8,9]. Iron stores are replenished through the phagocytosis of damaged RBCs by macrophages, which recycle iron. This process is also influenced by EPO, even though dietary intake typically compensates for most daily iron losses. Hepcidin plays a key role in regulating iron metabolism, affecting absorption and storage [9]. Produced by the liver and released by macrophages and adipocytes, hepcidin reduces iron absorption, promotes storage, and inhibits the proliferation of erythroid cells. Additionally, a decrease in GFR is linked to elevated hepcidin levels, as the kidneys are responsible for its elimination [8,10]. Hypoxia-inducible factor (HIF) is an essential transcription factor for erythropoiesis and iron metabolism. It consists of a stable β subunit and an oxygen-sensitive α subunit. Hypoxia response element-containing genes, which are controlled by prolyl-4-hydroxylase domain-containing proteins 1-3 (PHD1-3), are activated by HIF heterodimers. Under normal oxygen levels, HIF stabilizes and pairs with HIF-β, activating genes such as EPO and those involved in iron metabolism. Inhibiting PHDs may provide a potential treatment for anemia in CKD [10]. 

Anemia associated with CKD involves both absolute and functional iron deficiencies. Absolute iron deficiency may occur due to inadequate nutrition, reduced iron absorption, and losses from frequent blood draws. Functional iron deficiency, on the other hand, occurs when the body cannot effectively utilize its iron stores [11]. This type of anemia, often referred to as reticuloendothelial cell iron blockade, can result from any inflammatory condition, including CKD itself [11].

Diagnosis of IDA in CKD

Traditional biomarkers used to diagnose IDA include Hgb and hematocrit, reticulocyte count, mean corpuscular Hgb, and mean corpuscular volume. However, these parameters do not differentiate between absolute and functional IDA, as transferrin is increased in both cases [6-8,10]. Several biomarkers have been proposed to differentiate types of IDA in patients with CKD. Serum ferritin, soluble transferrin receptor (sTfR), percentage of hypochromic red blood cells (HRC%) and reticulocyte Hgb content (CHr), hepcidin, and plasma neutrophil gelatinase-associated lipocalin (NGAL) are some of the proposed biomarkers [8,12-14].

Serum ferritin is an acute-phase reactant that is frequently elevated in patients with CKD, irrespective of their iron stores. sTfR has been evaluated as a potential indicator of iron deficiency, but its use in IDA evaluation is limited due to testing requirements. Hepcidin has been evaluated as a marker of iron stores and iron responsiveness, but its correlation with ferritin levels is not reliable [8,9,12-14].

sTfR: Ferric iron is bound by the transferrin receptor, and the complex is then taken up by the cell. Then, erythroid progenitor cell membranes shed sTfR into the bloodstream. Therefore, it has been determined that sTfR may be a sign of iron insufficiency [8,12,13].

Hepcidin: Hepcidin, an essential hormone for iron metabolism, regulates iron absorption and storage. It is produced by the liver and released by macrophages and adipocytes. It decreases iron absorption, promotes iron storage, and inhibits erythroid cell proliferation. Due to its crucial role in the control of iron, hepcidin has been studied as a marker of iron responsiveness and storage, as well as a means of distinguishing between absolute and functional IDA [13].

HRC% and CHr: A number of additional indirect metrics have been taken into account while assessing IDA. To reflect the iron accessible for erythropoiesis in the recent timeline, HRC% and CHr both estimate the Hgb content in RBCs. Some have suggested using HRC% and CHr in the assessment of IDA in CKD because they are indicative of iron response [12,13].

NGAL has been associated with acute kidney injury, serving as an independent indicator of kidney disease progression and as a marker of inflammation. Additionally, NGAL plays a role in iron sequestration; in its free form, it can elevate extracellular iron levels, while in its bound form, it lowers them [12,13]. Table 1 summarizes the relationship between serum biomarkers and IDA.

**Table 1:** Relationship Between Serum Biomarkers and IDA IDA, iron-deficiency anemia; TSAT, transferrin saturation; CHr, reticulocyte Hgb content; HRC%, percentage of hypochromic red blood cells; sTfR, soluble transferrin receptor; NGAL, neutrophil gelatinase-associated lipocalin.

Serum Biomarkers	Relation to IDA
Ferritin	Decreases (<100 ng/dl diagnostic in non-dialysis-dependent)
TSAT	Decreases (<20% diagnostic)
CHr	Not reflective of stores
HRC%	Not reflective of stores
sTfR	May increase, can potentially note iron stores
Hepcidin	Increases
Plasma NGAL	Increases

Management of IDA in CKD

Diagnosing iron deficiency in individuals with CKD is crucial, as treatment can effectively address the related anemia. The standard care for managing IDA in CKD typically includes iron supplementation, either oral or intravenous, and/or ESA therapy [3,8,15-17].

Iron Therapy

A range of intravenous and oral iron formulations are available, each with specific advantages and disadvantages [3,8,15-17]. Due to variables like poor dietary iron absorption, frequent phlebotomy, persistent bleeding from platelet dysfunction brought on by uremia, and blood trapped in the dialysis machine, patients with renal disease are more likely to experience an iron shortage. Iron supplementation is crucial in treating anemia of CKD due to this deficit as well as the loss of circulating iron from erythropoiesis induced by ESAs. IV iron is the recommended option for hemolysis patients and those with advanced CKD because oral iron supplementation is generally ineffective due to high hepcidin levels. For individuals with CKD and anemia, KDIGO suggests a goal transferrin saturation (TSAT) of 20% to 30% and ferritin levels of 100 to 500 ng/ml [15]. IV iron with elevated ferritin or TSAT levels raises concerns because of the possibility of iron overload, which raises the risk of infection, oxidative stress damage, and tissue iron deposition. The possibility that some IV iron formulations may raise FGF23 levels as a result of interactions with the iron's surrounding carbohydrate shell is another serious concern. In clinical practice, new-generation IV iron compounds, including ferumoxytol, ferric isomaltose, and ferric carboxymaltose, are now often utilized. Their main benefit is the extremely stable carbohydrate shell, which allows for full replacement doses in just one or two infusions and inhibits the unchecked release of toxic-free iron. The polynuclear iron core in these agents is stable and has a low redox potential, which reduces the possibility of damaging oxidative stress events. This is another crucial characteristic [16].

Oral Iron Agents

Iron polysaccharides, ferrous fumarate, ferrous gluconate, ferrous sulfate, and ferric citrate (FC) are examples of oral agents [16].

FC serves as a phosphate binder in addition to being an IDA treatment FDA-approved for patients with CKD or ESRD. In the stomach's acidic environment, this molecule forms insoluble complexes with phosphates, while in the duodenum's alkaline environment, it releases ferric ions. Because of its dual function as a phosphate binder, the oral formulation of iron permits a more physiological response, potentially lowering the overall pill burden for patients. FC is just as effective as phosphate binders that are not based on calcium as well as those that are, according to studies. Furthermore, FC, independently of its effects on phosphorus reduction, decreases FGF23 levels in individuals on dialysis as well as those who are not. This may have important ramifications because elevated FGF23 levels are independently linked to anemia and cardiovascular death [12,18].

Ferric maltol is a new type of oral iron therapy that combines ferric iron with maltol, a naturally occurring sugar derivative, to form a stable complex. This formulation has both hydrophilic and lipophilic qualities, allowing bioavailable iron to be released in the digestive tract's neutral pH. Ferric iron, when taken orally, is complexed with maltol and reaches the intestinal mucosa, where it may be more readily absorbed by enterocytes than ferrous iron salts. It has been investigated for use in irritable bowel syndrome and has fewer gastrointestinal side effects since it avoids stomach metabolism. In the US and the EU, ferric maltol is licensed for the treatment of IDA [12].

Ferric pyrophosphate citrate, or FPC, was given FDA approval in 2015 to be used by dialysis patients. It is a complex iron salt that is water-soluble and free of carbohydrates and is given to hemolysis patients through dialysate. It may prevent iron sequestration in the reticuloendothelial macrophages by directly donating iron to transferrin. In patients with ESKD undergoing hemodialysis, FPC has been compared with placebo in the Continuous Replacement Using Iron Soluble Equivalents (CRUISE) 1 and 2 trials. When administered via dialysate, FPC was found to significantly lower ESA dosage while maintaining Hgb, TSAT, and ferritin more effectively than a placebo [12].

Liposomal/sucrosomial iron: Sucrosomes (a surfactant, sucrester, and other starch-like substances) form an extra layer around the ferric pyrophosphate core in lipid bilayers surrounding soluble iron. This reduces the possibility of adverse effects and avoids the downregulating effects of hepcidin by enabling iron to be taken up by microfold cells through the lymphatic system instead of the gastrointestinal tract. According to preliminary research, liposomal iron reduces the likelihood of side effects while improving Hgb in CKD patients. Sucrosomial iron has also been demonstrated to help patients with cancer, bariatric surgery patients, and celiac disease patients with their anemia. It has not been determined if sucrosomial iron is safe or effective in CKD patients [12].

Alternative to Iron Therapy

ESAs have been the mainstay of treatment for anemia in CKD patients. Enhancing quality of life, lowering cardiovascular morbidity, and improving survival have all been demonstrated when using ESAs to treat renal anemia [12,19]. A major contribution to improving anemia therapy has been made by darbepoetin alfa, the first ESA to provide longer dose intervals than EPO molecules, epoetins alfa, and beta. When Hgb levels fall below 10 g/dl in patients with CKD, ESAs are usually taken into consideration, according to Kidney Disease Improving Global Outcomes (KDIGO) guidelines. The rate of Hgb drop, response to iron therapy, symptoms of anemia, and the need for transfusions all play a role in the customized nature of ESA treatment. Darbepoetin alfa is normally dosed every two to four weeks, while EPO (50-100 units/kg IV or subcutaneously [SC]) is usually given every one to two weeks [12,19]. Regardless of dialysis requirement, the target Hgb utilizing ESAs is less than 11.5 g/dl in all CKD patients. The FDA has also released a warning on the use of ESAs to target Hb levels above 11 g/dl, which increases the risk of death, serious adverse cardiovascular events, and stroke. The possible impact of ESA use on cancer is a further worry. Because some neoplastic cells have EPO receptors expressed on them, administering ESA may cause these cells to proliferate more quickly. According to KDIGO guidelines, CKD patients with an active malignancy (grade 1B), a history of stroke (grade 1B), or a history of malignancy (grade 2C) should use ESAs with caution. The formation of anti-EPO antibodies and an allergic reaction are uncommon but serious side effects of using ESAs [20]. Immunosuppressive medications are typically used to treat this illness; however, stopping the ESA may be enough [21].

Limitations

This study has several limitations that should be explained. The review included studies from the past 15 years, which may exclude older, relevant studies. Biomarkers like ferritin, sTfR, and hepcidin were evaluated, but their diagnostic accuracy is limited. The review also highlighted the variability in treatment protocols, which may not be generalizable to all clinical settings. The lack of long-term data on the effects of iron supplementation and ESA use in CKD patients limits the ability to make definitive conclusions.

## Conclusions

There is a continuous opportunity to enhance the management of IDA in individuals with CKD, a condition that is often undertreated and linked to higher mortality and reduced quality of life.

Diagnosing iron deficiency in CKD patients is crucial, as treatment can effectively correct the anemia. Iron supplementation is commonly used to treat IDA in CKD, and it is important for the multidisciplinary care team to be aware of the specific considerations when administering this treatment. The multidisciplinary team plays a vital role in managing IDA in CKD, with the potential to significantly improve patient outcomes.
